# The Effects of Axitinib plus Tislelizumab in the Treatment of Advanced Renal Cell Carcinoma

**DOI:** 10.1155/2022/2700166

**Published:** 2022-03-24

**Authors:** Zhixin Wang, Jiaqi Chen, Qianyu Li, Na Li, Jing Yu, Feng Liu

**Affiliations:** ^1^Department of Urology, China-Japan Union Hospital of Jilin University, 126 Xiantai Street, Changchun 130033, China; ^2^Department of Nephrology, China-Japan Union Hospital of Jilin University, Changchun, China; ^3^Department of Nephrology, The Third Hospital of Jilin University, Changchun, China

## Abstract

**Objective:**

To study and analyze the clinical efficacy of axitinib combined with tislelizumab in the treatment of advanced renal cell carcinoma and its effects on renal function and serum cytokines.

**Methods:**

Totally 49 patients with advanced renal cancer treated in our hospital from November 2018 to January 2020 were randomized to treatment with axitinib (control group, *n* = 27) or axitinib combined with tislelizumab (study group, *n* = 22). The clinical efficacy, renal function and adverse reactions were compared between the two groups.

**Results:**

After treatment, both groups showed a significant decrease in blood urea nitrogen (BUN) and serum creatinine (SCR), but treatment with axitinib plus tislelizumab led to a significantly greater reduction than did the axitinib (each *p* < 0.05). After treatment, both groups showed a significant decrease in TNF-*β*1, VEGF, TIMP-1, and MMP-2, but treatment with axitinib plus tislelizumab led to a significantly greater reduction than did the axitinib (each *p* < 0.05). The study group had significantly higher rates of adverse reactions (*p* < 0.05). Significant difference was observed in ORR (59.1% vs 40.7%) and DCR (81.8% vs 66.7%) between the study group and the control group, with higher results in study group (*p* < 0.05). The study group was superior to the control group in OS (*p* < 0.05).

**Conclusion:**

Our study presents an effective alternative for advanced renal cell carcinoma by using axitinib plus tislelizumab. Limitations merit attention, and the study group had higher rates of adverse reactions. Therefore, further studies are suggested to secure a larger population of subjects.

## 1. Introduction

Renal cancer is one of the most common malignant tumors, and its prevalence ranks second to bladder cancer among urologic tumors, constituting about 3% to 5% of new cancer cases [1]. Nearly 50% of them died within 5 years after diagnosis. Renal cell carcinoma originates from renal epithelial cells and accounts for more than 90% of renal cancer, surgery is the mainstay for early local renal cell carcinoma [2]. However, many have progressed to the advanced stage or had distant metastasis due to the insidious symptoms, resulting in an inoperable condition. Some renal cancer recurred or metastasized after operation due to the incomplete resection, as a result neither radiotherapy and chemotherapy [3, 4], nor *a* interferon (INF-*α*) and interleukin-2 (IL-2) and other cytokines might be practical for the treatment. As medical technology advances, molecular-targeted drugs have emerged and generated a promising outcome [5, 6]. Axitinib, a multitarget tyrosine kinase inhibitor approved by the U.S. Food and Drug Administration (FDA), has been proven to be a potent drug for prolonging the progression-free survival (PFS) of renal cell carcinoma patients after systemic treatment failure [7, 8]. Cindilimab injection, toripalimab injection, carrelizumab injection, and tislelizumab injection recently approved for marketing in China belong to the third generation of humanized monoclonal antibody drugs. Tislelizumab is an immunotherapeutic drug targeting programmed cell death protein-1 (PD-1) [9, 10] and blocks the PD-1/death protein ligand 1 (PD-1/PD-L1) pathway by binding to the PD-1 receptor, thereby acting its therapeutic function [11]. It has currently been confirmed to be efficacious in the treatment of recurrent or refractory classical Hodge lymphoma by many authoritative official institutions [12−14], yet, few studies have ever been carried out on the treatment of renal cancer. In light of this, the present study intended to investigate the combination of axitinib combined with tislelizumab in the treatment of renal cancer aiming to provide an experimental basis for the future scheme formulation.

## 2. Materials and Methods

### 2.1. Study Design and Participants

Totally 49 patients with advanced renal cancer treated in our hospital from November 2018 to January 2020 were randomized to treatment with axitinib (control group, *n* = 27) or axitinib combined with tislelizumab (study group, *n* = 22). In the study group, there were 15 males and 7 females, aged 53∼71, with an average age of (63.25 ± 5.40) years; tumor diameter was 1.28∼8.12 cm with an average tumor diameter of (4.55 ± 0.47) cm. In the control group, there were 16 males and 6 females, aged 52∼70, with an average age of (62.4 ± 5.54) years; tumor diameter was1.27∼8.30 cm with an average tumor diameter of (4.58 ± 0.49) cm. The baseline data of the two groups were well balanced and homogeneous (*p* > 0.05). Inclusion criteria are as follows: (1) advanced renal cell carcinoma with clear cell carcinoma was diagnosed histologically; (2) the interval from radiotherapy or operation was ≥4 weeks; (3) the estimated survival time was >3 months, and the score of ECoG was 0∼1; (4) there were measurable lesions according to the Response Evaluation Criteria in Solid Tumors (RECIST); and (5) complete clinical data and no transfer or discharge halfway. Exclusion criteria are as follows: (1) known brain metastasis; (2) patients with serious adverse drug reactions during treatment and intolerance; (3) patients with bleeding tendency or undergoing thrombolytic or anticoagulant treatment; (4) patients with myocardial ischemia or myocardial infarction> grade I, arrhythmia and grade I cardiac insufficiency; (5) arteriovenous thrombosis occurred within 6 months; and (6) patients with significant hepatic diseases. The participants, who agreed to participate in the study, were provided with an informed document about the purposes of the study. Their consent on participating in the study was voluntarily signed by participants. All the procedures were in strict accordance with the protocol of ethics committee of our hospital.

### 2.2. Interventions

The control group received axitinib (specification: 5 mg/tablet, batch number: 20191123, approval number: h20150221, Pfizer manufacturing Deutschland GmbH), orally twice a day. After two consecutive weeks of administration, it can be increased to 7 mg each time within the safety threshold. After another two consecutive weeks of administration, it can be further increased to a maximum of 10 mg each time, twice a day. In cases of adverse reactions, the dose shall be adjusted according to its severity. The study group was additionally given tislelizumab injection (specification: 10 ml/100 mg, batch no. 20200108, approval no. s20190045, Baiji Shenzhou (Shanghai) Biotechnology Co., Ltd.) 200 mg intravenously once every 3 weeks. The dose is adjusted depending on the hematological or nonhematological toxicity during the treatment, and the treatment would be discontinued until the tumor progression or withdrawal occurs due to the life-threatening toxic reactions.

### 2.3. Outcome Measures

The efficacy is evaluated using imaging examination and reexamination of CT or MRI after 2 cycles (6 weeks) of treatment, and by referring to RECIST (version 1.1), and categorized as complete response (CR), partial response (PR), stable disease (SD) and progressive disease (PD). Objective response rate (ORR) = (CR cases + PR cases)/total cases × 100% and disease control rate (DCR) = (CR cases + PR cases + SD cases)/total cases × 100%.

Three ml of peripheral venous blood were collected before and after treatment, and the supernatant was centrifuged for the determination of tumor necrosis factor-*β*1 (TNF-*β*1), vascular endothelial growth factor (VEGF), tissue inhibitor of metalloproteinase-1 (TIMP-1), and matrix metalloproteinase-2 (MMP-2) by using the enzyme-linked immunosorbent assay (ELISA) kits produced by Biyuntian Biotechnology Co., Ltd. BIOBASE cryogenic centrifuge was produced by Shandong Boke Scientific Instrument Co., Ltd. PRIME60 automatic biochemical analyzer was produced by Thermo Fisher Technology Co., Ltd.

Adverse drug reactions were appraised in accordance with the international standard for Common Terminology Criteria for Adverse Events (CTCAE) version 5.0 [15]. Symptomatic treatment was mainly used in case of grade 1∼2 adverse reactions. In case of adverse reactions with grade 3 and above, the drug should be discontinued or targeted intervention measures should be taken.

All subjects were required to be rechecked once a month within 40 months after treatment. Follow-up was conducted via telephone to determine survival. Progression-free survival (PFS) is defined as the time from the treatment to the tumor growth, and the nonprogression at the time of the end of follow-up is counted as 1 year; overall survival (OS) is defined as the time from the beginning of treatment to the patient's death, and it is counted as 1 year at the time of the end of follow-up.

### 2.4. Statistical Analysis

All data analysis was performed with SPSS 23.0. The enumeration data and measurement data were expressed as percentage (%) and (*x* ± *s*), and examined by the chi-square test, and *t*-test. The graphics were plotted by GraphPad Prism 8. The conventional *p* < 0.05 was used to assess statistical significance.

## 3. Results

### 3.1. Renal Function

After treatment, both groups showed a significant decrease in blood urea nitrogen (BUN) and serum creatinine (SCR), but treatment with axitinib plus tislelizumab led to a significantly greater reduction than did the axitinib (each *p* < 0.05, Table 1).

### 3.2. Serum Cytokine

After treatment, both groups showed a significant decrease in TNF- *β* 1, VEGF, TIMP-1, and MMP-2, but treatment with axitinib plus tislelizumab led to a significantly greater reduction than did the axitinib (each *p* < 0.05, Table 2).

### 3.3. Adverse Reactions between the Two Groups

The study group had significantly higher rates of adverse reactions (*p* < 0.05, Table 3).

### 3.4. Treatment Effects

A significant difference was observed in ORR (59.1% vs 40.7%) and DCR (81.8% vs 66.7%) between the study group and the control group, with higher results in the study group (*p* < 0.05, Table 4).

### 3.5. PFS

**Figure 1:**
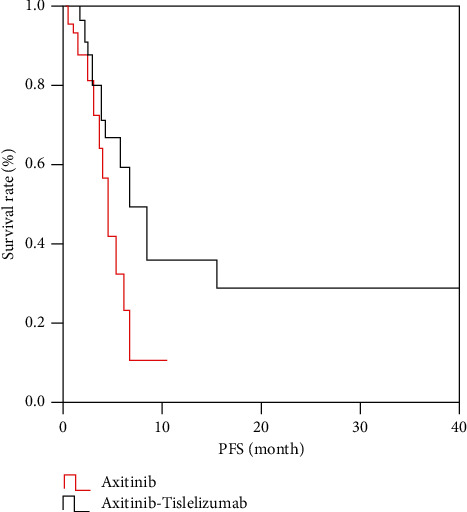
Comparison of PFS between the study group and control group.

The longest follow-up time of all patients was 40.0 months, and the median follow-up time was 7.5 months. The PFS of the study group was 1.0∼40.0 months, and the median PFS was 7.0 months. The PFS of the control group was 2.0∼12.0 months, and the median PFS was 4.7 months. The PFS of the study group was higher than that of the control group (*p* < 0.05), as shown in Figure 1.

### 3.6. OS

**Figure 2:**
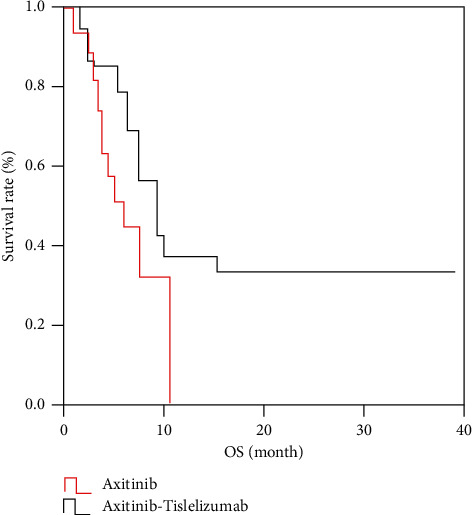
Comparison of OS between the study group and control group.

The OS of the study group was 1.0∼40.0 months, and the median OS was 8.9 months; The OS of the control group was 2.0∼12.0 months, and the median OS was 5.8 months. The study group was superior to the control group in OS (*p* < 0.05), as shown in Figure 2.

## 4. Discussion

Renal cancer is a common malignant solid tumor in urology [16], the annual new cases exceed 295,000, and the renal cancer-related deaths reach 134,000. With the in-depth study of the pathogenesis of renal cancer, a variety of molecular-targeted drugs have been widely used in the treatment of advanced renal cell carcinoma and proven to be efficacious [4]. Axitinib, developed by Pfizer, is a second-generation VEGFR inhibitor [17] and was approved by the U.S. FDA in January 2012. It selectively acts on VEGFR1, VEGFR2, and VEGFR3 and inhibits tumor growth by inhibiting VEGF-mediated endothelial cell proliferation and survival [18, 19]. Tislelizumab, a monoclonal antibody with high affinity and specificity for PD-1, belongs to tumor immune drugs called immune checkpoint inhibitors. Given the absence of research on the clinical application of axitinib plus tislelizumab in the treatment of advanced renal cell carcinoma, this study aims to explore the effects of axitinib in combination with tislelizumab on renal function, serum factors, and clinical efficacy.

The *t*-test in the present study revealed a significant reduction of BUN, SCR, TNF-*β*1, VEGF, TIMP-1, and MMP-2 after treatment, and the use of axitinib plus tislelizumab resulted in greater reduction, indicating that axitinib combined with tislelizumab can improve renal function, probably because the interplay of these two plays a central role in reducing tumor blood supply via blocking the formation of neovascularization, so as to inhibit the proliferation and growth of VEGF, reduce the angiogenesis for tumor nutrients provision, and inhibit the occurrence and growth of tumor cells [20]. Moreover, the tislelizumab immunotherapy drug binding to PD-1 on the tumor surface stimulates lymphocyte secretion, boosts the resistance to tumor cells, attenuates or even reverses T cell disability or failure, reactivates the attack and killing ability of effector T cells, and enhances tumor resistance [21]. The action of the combination can restore the renal function accordingly. The study group demonstrated a superior performance versus the control group in clinical efficacy, with more adverse reactions in the study group, indicating that axitinib plus tislelizumab in the treatment of renal cell carcinoma can effectively improve the treatment efficiency, with an inferior safety profile. Encouragingly, similar to the results of Turajlic [22], in the present trial, the study group outperformed the control group with respect to the PFS and OS.

Our study presents an effective alternative for advanced renal cell carcinoma by using axitinib plus tislelizumab, but limitations merit attention. Given the overall difference in adverse reactions, we hypothesize that this resulted from the small sample size, which would possibly bias our results toward the null. Therefore, further studies are suggested to secure a larger population of subjects.

## Figures and Tables

**Table 1:** Renal function (*x* ± *s*).

Groups	*n*	BUN		SCr	
		Before treatment	After treatment	Before treatment	After treatment

Study group	22	17.44 ± 0.87	3.27 ± 0.52	114.24 ± 13.57	64.81 ± 6.19
Control group	27	17.21 ± 0.83	5.14 ± 0.53	114.13 ± 13.26	78.36 ± 7.13
*t*		0.944	12.388	0.029	7.622
*p* value		0.35	<0.001	0.977	<0.001

**Table 2:** Serum cytokine (*x* ± *s*).

		Study group(*n* = 22)	Control group(*n* = 27)	*t*	*p* value

TNF-*β*1	Before treatment	45.33 ± 5.17	45.48 ± 5.29	0.048	0.962
	After treatment	21.90 ± 3.62^*∗*^	31.77 ± 4.02^*∗*^	9.934	<0.001
VEGF	Before treatment	38.67 ± 4.95	37.99 ± 4.97	0.477	0.636
	After treatment	16.36 ± 2.77^*∗*^	22.73 ± 2.98^*∗*^	7.679	<0.001
TIMP-1	Before treatment	82.39 ± 8.51	83.02 ± 8.86	0.252	0.802
	After treatment	36.04 ± 5.82^*∗*^	47.11 ± 6.54^*∗*^	6.188	<0.001
MMP-2	Before treatment	185.36 ± 26.62	186.34 ± 27.03	0.127	0.899
	After treatment	57.67 ± 5.39^*∗*^	76.47 ± 7.98^*∗*^	9.427	<0.001

^*∗*^The *p* value for the difference between the study group and the control group is <0.05.

**Table 3:** Adverse reactions between the two groups (*n* (%)).

	Study group(*n* = 22)			Total	Control group (*n* = 27)			Total
	I	II	III	60	I	II	III	54
Hypertension	6	3	1	10	5	5	1	11
Hoarseness	5	5	1	10	4	3	0	7
Hypothyroidism	1	1	0	2	1	0	0	1
Anorexia	3	2	0	5	3	3	0	6
Fatigue	5	4	0	9	4	3	0	7
Diarrhea	3	3	0	6	2	2	0	4
Hand-foot skin reaction	4	2	0	6	3	1	0	4
Immune associated pneumonia	2	1	0	3	2	1	0	3
Vomiting	5	4	0	9	6	5	0	11

**Table 4:** Treatment effects (*n* (%)).

Groups	*n*	CR	PR	SD	PD	ORR	DCR

Study group	22	0	13	5	4	59.1	81.8
Control group	27	0	11	7	9	40.7	66.7
*X* ^2^						6.771	5.963
*p* value						0.009	0.015

## Data Availability

The datasets used during the present study are available from the corresponding author upon reasonable request.
